# Prognostic Role of Adjuvant Chemotherapy in Node-Negative (N0), Triple-Negative (TN), Medullary Breast Cancer (MBC) in the Korean Population

**DOI:** 10.1371/journal.pone.0140208

**Published:** 2015-11-12

**Authors:** SeungTaek Lim, Se Ho Park, Heong Kyu Park, Min Hee Hur, Se Jeong Oh, Young Jin Suh

**Affiliations:** 1 Division of Breast & Thyroid Surgical Oncology, Department of Surgery, College of Medicine, St. Vincent’s Hospital, The Catholic University of Korea, Jungbu-daero 93, Paldal-gu, Suwon-si, Gyeonggi-do, 442-723, Republic of Korea; 2 Department of Surgery, Yonsei University College of Medicine, 50 Yonsei-ro, Seodaemun-gu, Seoul, 120-752, Republic of Korea; 3 Breast Cancer Center, Department of Surgery, Gachon University Gill Hospital, 1198, Guwoldong, Incheon, 405-760, Republic of Korea; 4 Department of Surgery, Cheil General Hospital and Women`s Healthcare Center, Dankook University College of Medicine, 17 Seoae-ro 1-gil, Jung-gu, Seoul, 100-380, Republic of Korea; 5 Department of Surgery, Incheon St. Mary`s Hospital, College of Medicine, The Catholic University of Korea, 56 Dongsu-ro, Bupyung-gu, Incheon, 403-720, Republic of Korea; University of North Carolina School of Medicine, UNITED STATES

## Abstract

**Background:**

Despite the favorable prognosis for medullary breast cancer (MBC), the guidelines for the use of adjuvant chemotherapy for MBC have not been clearly established. This study investigated the prognostic role of adjuvant chemotherapy in Korean patients with node-negative (N0), triple-negative (TN) MBC patients.

**Methods:**

We included data from 252 patients with N0 TN MBC, obtained from the Korean Breast Cancer Registry database. Patients were categorized as those who did not undergo adjuvant chemotherapy (group I) or those who did (group II). Clinicopathological characteristics, breast cancer-specific survival (BCSS), and overall survival (OS) were compared between the groups. In addition, a subgroup analysis for survival based on tumor size was conducted.

**Results:**

A total of 252 N0 TN MBC patients with tumor sizes >1 cm who were diagnosed between April 1997 and March 2011 were enrolled. The median age was 44.95 years (range, 25–72 years), and the median follow-up period was 93.94 months (range, 23–195 months). Overall, the BCSS and OS in group II (97.3% and 97.3%, respectively) were significantly better compared with those in group I (89.2% and 86.2%, respectively). In the subgroup analysis, in patients with tumors >2 cm in size, those in group II had significant better BCSS and OS (97.5% and 97.5%, respectively) compared with those in group I (78.3% and 73.9%, respectively). In contrast in those with tumors 1–2 cm in size, there were no significant differences in BCSS and OS between the groups (both 97.1% for group I, and 95.2% and 92.9%, respectively for group II). Multivariate analysis revealed that adjuvant chemotherapy significantly improved BCSS (P = 0.009) and OS (P = 0.007), but only for patients with larger tumors (>2 cm).

**Conclusions:**

In patients with N0 TN MBC, adjuvant chemotherapy had a significant clinical survival benefit, but only in those with tumors >2 cm.

## Introduction

Medullary breast cancer (MBC), which was first described by Ridolfi et al. in 1977 [1], is a rare histologic breast cancer subtype that accounts for 1.1–7% of all invasive breast cancers [2–5]. Histologically, the tumor is characterized by medullary growth of large cells with a high histological grade with a particularly high mitotic count, well-circumscribed edges, central fibrosis and necrosis, and the frequent presence of lymphocytic infiltration [6].

Triple-negative (TN) breast cancer describes a molecular subtype of breast cancer in which estrogen receptor (ER), progesterone receptor (PR), and human epidermal growth factor receptor-2 (HER2) expressions are negative; it accounts for 15% of all breast cancers [7]. TN breast cancer has been associated with a poor prognosis, and possess typically aggressive characteristics such as younger age at diagnosis and higher grade [8].

Previous studies have reported that ~70–90% of MBC cases harbor the TN molecular subtype [9–16]. Because of the lack of targeted therapy, the mainstream adjuvant therapy for TN breast cancer is systemic chemotherapy, and because of the poor patient prognosis and increased sensitivity of TN breast cancer to chemotherapy, on average patients with TN breast cancer are likely to undergo more intensive chemotherapy regimens [17,18]. Currently, most clinicians apply the same guidelines for adjuvant chemotherapy to TN MBC and TN invasive ductal carcinoma (IDC). However, although MBC has been associated with larger tumor size, higher grade, and an increased proportion of hormone receptor negativity compared with IDC [19–21], recent studies indicated that MBC had a better prognosis compared with IDC [22,23].

This raises the question of whether it is reasonable to adopt the adjuvant chemotherapy regimens used for IDC to MBC. Furthermore, there is no consensus between current guidelines on the necessity of adjuvant chemotherapy for patients with TN MBC, especially for those with early stage disease. Therefore, in the present study, we evaluated the prognostic role of adjuvant chemotherapy for Korean patients with node negative (N0), TN MBC whose tumors were >1 cm in size by using the Korean Breast Cancer Registry (KBCR) database. In addition, we performed a subgroup analysis according to tumor size, to determine the effective criterion for adjuvant chemotherapy in N0 TN MBC.

## Methods

### Ethics statement

This study was approved by the Institutional Review Board of St. Vincent Hospital. All participants in this study provided written informed consent for storage of their medical information in the database and for research use of this information.

### The Korean Breast Cancer Registry

The KBCR database is a nationwide database that includes 41 university hospitals and 61 surgical training hospitals [24]. This database provides information pertaining to patient survival, sex, age, the surgical method used, the stage of cancer based on the 7^th^ American Joint Committee on Cancer classification, the pathological characteristics of the tumor, and any adjuvant treatment received.

### Study population

We retrospectively reviewed the clinicopathological data of female patients in the KBCR database who were diagnosed with invasive breast cancer between April 1997 and March 2011. Among 74,969 patients with invasive breast cancer, we identified 755 with MBC. In sequence, we selected the patients who had a verified tumor size >1 cm, no axillary lymph node metastasis, and the TN molecular subtype. Patients were excluded if they had metastatic disease at the time of diagnosis, multifocal or multicentric breast cancer, a history of previous ipsilateral or contralateral breast cancer, or if they had undergone neoadjuvant chemotherapy. Patients who were not treated with a curative intent, those without follow-up data, and those whose hormone receptor status was unknown were also excluded. A fluorescence in situ hybridization (FISH) assay was used to confirm the HER2 status if there was indeterminate (2+) immunohistochemical results for HER2/neu staining. Finally, 252 patients were included in this study.

### Clinicopathological characteristics and treatment regimens

Clinicopathological variables including age at diagnosis, type of surgery, tumor size, number of harvested lymph nodes, histologic grade, lymphatic invasion, vascular invasion, and status of adjuvant treatment (radiotherapy and chemotherapy) were evaluated.

All patients considered to be at risk for relapse received adjuvant chemotherapy with or without subsequent radiotherapy based on the local clinician`s discretion. Patients were classified into two groups according to the status of adjuvant chemotherapy: group I, those who did not receive adjuvant chemotherapy (n = 65); and group II, those who received adjuvant chemotherapy (n = 187). In group II, all patients completed full cycle of the chosen chemotherapy regimen administered following a standard protocol.

Administered chemotherapy regimens were anthracycline-based (6 cycles of doxorubicin + cyclophosphamide [AC] or epirubicin+ cyclophosphamide [EC] or fluorouracil + doxorubicin + cyclophosphamide [FAC] or fluorouracil + epirubicin + cyclophosphamide [FEC]), or non-anthracycline-based (6 cycles of cyclophosphamide + methotrexate + fluorouracil [CMF]), or anthracycline plus taxane-based (6 cycles of paclitaxel + doxorubicin [TA] or paclitaxel +doxorubicin + cyclophosphamide [TAC] or paclitaxel +epirubicin+ cyclophosphamide [TEC]). All chemotherapy regimens were administered in a concurrent manner, except in 1 patient who underwent TAC chemotherapy (4 cycles of AC, followed by 4 cycles of T).

### Survival analysis

We evaluated the association between adjuvant chemotherapy and breast cancer-specific survival (BCSS), and overall survival (OS). BCSS was defined as the period from the date of breast cancer diagnosis until the date of breast cancer-related death or the date of last follow-up. OS was defined as the period from the date of breast cancer diagnosis until the date of death from any cause or the date of last follow-up.

### Statistical analyses

Characteristic differences between the groups were compared using the independent t-test, Pearson`s chi-square test, or Fisher`s exact test. Survival curves were obtained using the Kaplan-Meier method, and were compared using the log rank test. Multivariate analyses were conducted using Cox`s proportional hazards model to assess the prognostic significance of adjuvant chemotherapy, and hazard ratios (HRs) and 95% confidence intervals (95% CIs) were estimated for each variable. Multivariate models were adjusted for age at diagnosis, surgery type, number of harvested lymph nodes, lymphatic invasion, vascular invasion, histologic grade, radiotherapy, and chemotherapy. All statistical tests were two-sided, and the statistical significance was set at *P*<0.05. All statistical analyses were performed using SPSS Statistics for windows, Version 12.0 (SPSS Inc., Chicago, IL, USA).

## Results

### Patient and tumor characteristics

The baseline characteristics of the study population and the subgroups are shown in Table 1.

**Table 1:** Baseline patient characteristics.

	Overall patients (n = 252)			Tumor size 1–2 cm (n = 110)			Tumor size >2 cm (n = 142)		
	Group I (n = 65)	Group II (n = 187)		Group I (n = 42)	Group II (n = 68)		Group I (n = 23)	Group II (n = 119)	
	No. (%)	No. (%)	*P*-value	No. (%)	No. (%)	*P*-value	No. (%)	No. (%)	*P*-value
Age (years)									
Mean ± SD	45.23±10.35	44.86±8.88	0.779	46.43±10.09	43.94±8.42	0.166	43.04±10.68	45.38±9.13	0.277
≤50	47 (72.3%)	132 (73.7%)	0.792	30 (71.4%)	53 (77.9%)	0.441	17 (73.9%)	79 (66.4%)	0.480
>50	18 (27.7%)	55 (29.4%)		12 (28.6%)	15 (22.1%)		6 (26.1%)	40 (33.6%)	
Surgery									
BCS	49 (75.4%)	131 (70.1%)	0.412	34 (81.0%)	58 (85.3%)	0.550	15 (65.2%)	73 (61.3%)	0.726
MRM	16 (24.6%)	56 (29.9%)		8 (19.0%)	10 (14.7%)		8 (34.8%)	46 (38.7%)	
Tumor size (cm)									
Mean ± SD	2.18±0.94	2.52±1.12	0.027	1.68±0.26	1.69±0.29	0.921	3.08±1.08	3.00±1.14	0.768
1–2	42 (64.6%)	68 (36.4%)	<0.001	42 (100%)	68 (100%)	-	0 (0%)	0 (0%)	0.616
2–5	21 (32.3%)	113 (60.4%)		0 (0%)	0 (0%)		21 (91.3%)	113 (95.0%)	
>5	2 (3.1%)	6 (3.2%)		0 (0%)	0 (0%)		2 (8.7%)	6 (5.0%)	
Number of harvested LN	14.49±7.64	14.67±9.21	0.887	12.12±6.19	12.94±9.05	0.573	18.83±8.25	15.66±9.20	0.128
≤13	29 (44.6%)	89 (47.6%)	0.678	22 (52.4%)	43 (63.2%)	0.261	7 (30.4%)	46 (38.7%)	0.456
>13	36 (55.4%)	98 (52.4%)		20 (47.6%)	25 (36.8%)		16 (69.6%)	73 (61.3%)	
Histologic grade									
1–2	30 (46.2%)	84 (44.9%)	0.863	15 (35.7%)	29 (42.6%)	0.471	15 (65.2%)	55 (46.2%)	0.095
3	35 (53.8%)	103 (55.1%)		27 (64.3%)	39 (57.4%)		8 (34.8%)	64 (53.8%)	
Lymphatic invasion									
No	60 (92.3%)	171 (91.4%)	0.828	38 (90.5%)	57 (83.8%)	0.323	22 (95.7%)	114 (95.8%)	0.975
Yes	5 (7.7%)	16 (8.6%)		4 (9.5%)	11 (16.2%)		1 (4.3%)	5 (4.2%)	
Vascular invasion									
No	61 (93.8%)	178 (95.2%)	0.746	39 (92.9%)	63 (92.6%)	0.967	22 (95.7%)	115 (96.6%)	0.814
Yes	4 (6.2%)	9 (4.8%)		3 (7.1%)	5 (7.4%)		1 (4.3%)	4 (3.4%)	
RTx.									
No	19 (29.2%)	57 (30.5%)	0.850	11 (26.2%)	9 (13.2%)	0.087	8 (34.8%)	48 (40.3%)	0.618
Yes	46 (70.8%)	130 (69.5%)		31 (73.8%)	59 (86.8%)		15 (65.2%)	71 (59.7%)	

BCS, breast-conserving surgery; LN, lymph node; MRM, modified radical mastectomy; SD, standard deviation; RTx, radiotherapy. Group I, patients who did not receive chemotherapy; Group II, patients who received chemotherapy.

The mean patient age for the whole cohort was 44.95 years (range, 25–72 years). Of the 252 patients, 180 underwent breast-conserving surgery (including 161 with axillary dissection and 19 with sentinel lymph node biopsy alone) and 72 underwent a modified radical mastectomy (including 68 with axillary dissection and 4 with sentinel lymph node biopsy alone). The proportion of patients with tumor sizes 2–5 cm was significantly higher in group II compared with group I (*P*<0.001). Otherwise, there were no significant differences between groups. For the subgroup analysis, the clinicopathological characteristics of patients were also not different between group I and group II or each subgroup (tumor size 1–2 cm and >2 cm).

The chemotherapy regimens administered to patients in Group II are shown in Table 2.

**Table 2:** Chemotherapy regimens for patients who received adjuvant chemotherapy.

	Overall patents (n = 187)	Tumor size 1–2 cm (n = 68)	Tumor size >2 cm (n = 119)	
	No. (%)	No. (%)	No. (%)	*P*-value
Anthracycline-based	118 (63.1)	43 (63.2)	75 (63.0)	0.983
Non-anthracycline based	64 (34.2)	23 (33.8)	41 (34.5)	
Anthracycline + Taxane	5 (2.7)	2 (2.9)	3 (2.5)	

Anthracycline-based: AC,EC,FAC,FEC; Non-anthracycline-based: CMF; Anthracycline + Taxane-based: TA,TAC,TEC

In total, 118 patients (63.1%) received anthracycline-based chemotherapy (containing 58 AC, 7 EC, 29 FAC, 24 FEC), 64 patients (34.2%) received non-anthracycline-based chemotherapy (containing 64 CMF), and 5 patients (2.7%) received anthracycline plus taxane-based chemotherapy (containing 3 TA, 1 TAC, 1 TEC). The proportion of the applied chemotherapy regimens was not significantly different between the subgroups (*P* = 0.983).

### Follow-up and patient outcomes

**Fig 1:**
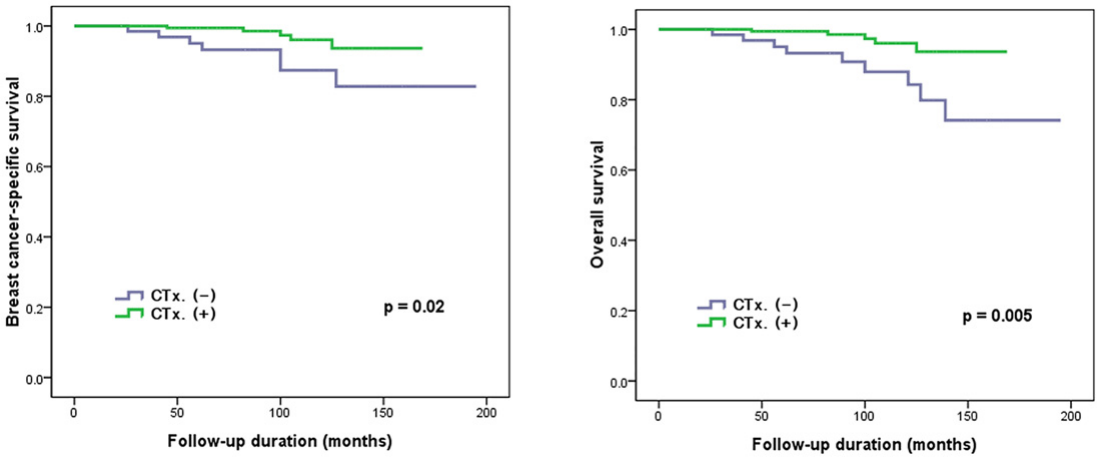
The association between adjuvant chemotherapy (CTx.) and survival (breast cancer-specific survival and overall survival) in all patients.

The median follow-up time was 96 months (range, 23–195 months). During follow-up, 14 patients died including 12 who died of breast cancer-related causes. For the whole population, the BCSS was 95.2% and the OS was 94.4%. The BCSS and OS for group II (97.3% and 97.3%, respectively) were significantly better compared with those for group I (89.2% and 86.2%, respectively) (Fig 1).

**Fig 2:**
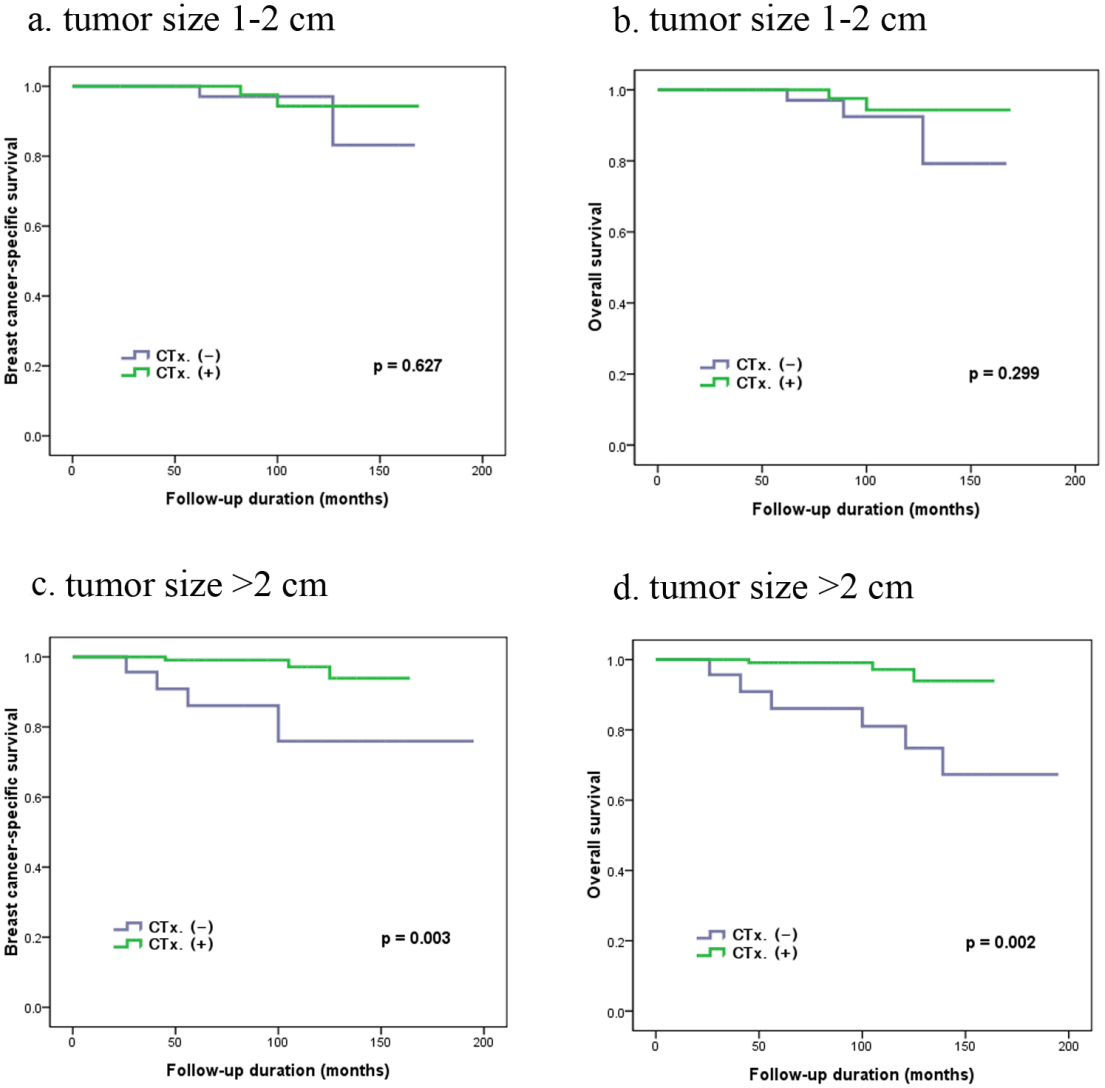
The association between adjuvant chemotherapy (CTx.) and survival (breast cancer-specific survival and overall survival) in the subgroup analysis according to tumor size (a,b) patients with tumor size 1–2 cm (c,d) patients with tumor size >2 cm.

For the subgroup analysis, in patients with larger tumors (>2 cm), those in group II had a significant better BCSS and OS (97.5% and 97.5%, respectively) compared with those in group I (78.3% and 73.9%, respectively) (Fig 2). In patients with tumors 1–2 cm in size, there was no significant differences in BCSS or OS between group I (97.1% and 97.1%, respectively) and group II (95.2% and 92.9%, respectively) (Fig 2).

### Multivariate survival analysis

Multivariate analyses to determine the prognostic impact of adjuvant chemotherapy for the overall study population and subgroups are shown in Table 3.

**Table 3:** Multivariate analysis evaluating the survival impact of adjuvant chemotherapy.

	Breast cancer-specific survival		Overall survival	
	Adjusted HR (95% CI)	*P*-value	Adjusted HR (95% CI)	*P*-value
All patients				
Group I	Reference		Reference	
Group II	0.279 (0.088–0.880)	0.029	0.233 (0.078–0.702)	0.01
Tumor size 1–2 cm				
Group I	Reference		Reference	
Group II	0.419 (0.047–3.729)	0.436	0.230 (0.029–1.799)	0.161
Tumor size >2 cm				
Group I	Reference		Reference	
Group II	0.146 (0.034–0.622)	0.009	0.141 (0.034–0.583)	0.007

HR, hazard ratio; CI, confidence interval. Group I, patients who did not receive adjuvant chemotherapy; Group II, patients who received adjuvant chemotherapy. Adjusted for age, surgery type, number of harvested lymph node, lymphatic invasion, vascular invasion, histologic grade, radiotherapy, and chemotherapy.

Adjuvant chemotherapy significantly improved BCSS (*P* = 0.029) and OS(*P* = 0.01) in the whole study population. For the subgroup analysis, chemotherapy significantly improved BCSS (*P* = 0.009) and OS (*P* = 0.007), but only in those with larger tumors (>2 cm). For those with smaller tumors, adjuvant chemotherapy did not improve BCSS (*P* = 0.436) or OS (*P* = 0.161).

## Discussion

In this study, we evaluated the prognostic role of adjuvant chemotherapy in patients with N0 TN MBC. We demonstrated that adjuvant chemotherapy significantly improved BCSS and OS in patients with N0 TN MBC, but only in those with larger tumors (>2cm).

The proportion of patients in the KBCR that we identified as having MBC (1%) was concordant with the percentage reported in previous studies [22]. In addition, the mean patient age for those with N0 TN MBC in the present study was less compared with that reported previously for patients with N0 TN IDC [25]. In agreement with this finding, Park et al. previously reported that patients with MBC were significantly younger compared to those with IDC [26]. Furthermore, Chu et al. demonstrated that patients with TN MBC tended to be younger compared with patients with other molecular MBC subtypes [27]. Consequently, these studies imply that the relatively young mean age of our study population is not so surprising when considering the combination of medullary histology and TN molecular subtype.

Patients with MBC have been reported to harbor larger tumors compared to patients with IDC [19]. However, when limited to N0 TN patients, there was little evidence of differences in tumor size between patients with MBC and those with IDC. In patients with IDC, Munzone et al. [7] showed that the proportion of patients with tumor sizes >2 cm in those with N0 TN IDC was 36.5% (181/496), whereas Wu et al.[28] reported that 47.8% (132/276) of N0 TN IDC patients in their study had tumors >2 cm in size. In our study, the proportion of N0 TN MBC patients with tumor sizes >2 cm was 56.3% (142/252). Our results indicate that the characteristics of MBC (larger tumor sizes than IDC) were maintained even in patients with N0 status and TN molecular subtype.

The putative adverse effects of systemic chemotherapy can significantly impair patient quality of life; therefore, chemotherapy should be considered only for patients in whom a definite prognostic benefit is likely. Currently, there are no specific guidelines for adjuvant chemotherapy in patients with MBC, most likely because of the low MBC incidence rate and lack of large-scale studies. Therefore, as mentioned earlier, adjuvant chemotherapy for these patients is mostly based on that administered for IDC. However, recent studies have reported that MBC has a superior prognosis compared with IDC [22,23], suggesting the necessity for independent guidelines for adjuvant chemotherapy in patients with MBC.

In Korea, many clinicians refer to the National Comprehensive Cancer Network (NCCN) or St. Gallen guidelines to determine the treatment strategy involving adjuvant chemotherapy. However, there is no agreement between these guidelines for adjuvant chemotherapy in N0 TN MBC patients with tumor sizes >1cm. For N0 TN MBC, the NCCN guideline recommends adjuvant chemotherapy as evidence category 1 when the tumor size was >1 cm [29], whereas the St. Gallen guideline does not recommend adjuvant chemotherapy for N0 TN MBC regardless of tumor size [30]. Considering that a large proportion of patients with MBC were N0, TN, and tumors >1 cm, these guidelines significantly hamper physician decision-making. In the present study, we showed that adjuvant chemotherapy had significant survival benefits in patients with N0 TN MBC whose tumor size was >2 cm, but not in those with smaller tumors. Therefore, following the NCCN guidelines wound mean that those in our population with tumors sized 1–2 cm would receive unnecessary treatment, whereas according to the St. Gallen guidelines, those with tumors >2 cm would not receive sufficient treatment. These findings provide evidence for a proposal to formulate specific guidelines for adjuvant chemotherapy in patients with N0 TN MBC.

The present study has several limitations. First, we did not investigate detailed histologic subtypes of MBC (typical or atypical) because the KBCR database does not contain such information. Although we cannot rule out that this may have influenced our findings, Rakha et al. have previously reported a similar prognostic outcome between typical and atypical MBC [31]. Secondly, because the KBCR database is largely composed of local reports, it was difficult to ensure the quality and reliability of available data. Lastly, we could not perform survival analysis for recurrence, because recurrence data is not available in the KBCR database. Despite these limitations, this was the first study to investigate the prognostic role of adjuvant chemotherapy in patients with N0 TN MBC using a relative large study population with homogenous clinicopathological characteristics.

## Conclusions

In conclusion, we demonstrated that adjuvant chemotherapy had significant survival benefits in Korean N0 TN MBC patients with tumors>2cm. These results suggest that adjuvant chemotherapy could possibly be omitted in N0 TN MBC patients with smaller tumors, and they provide evidence to support the development of specific guidelines for the use of adjuvant chemotherapy in patients with N0 TN MBC.
